# Sodium and potassium disorders in patients with COPD exacerbation presenting to the emergency department

**DOI:** 10.1186/s12873-022-00607-7

**Published:** 2022-03-24

**Authors:** Gregor Lindner, Stefano Herschmann, Georg-Christian Funk, Aristomenis K. Exadaktylos, Rebecca Gygli, Svenja Ravioli

**Affiliations:** 1 Department of Internal and Emergency Medicine, Buergerspital Solothurn, Solothurn Switzerland; 2 Karl-Landsteiner-Institute for Lung Research and Pulmonary Oncology, Wilheminenspital, Vienna, Austria; 3 Department of Emergency Medicine, Inselspital, University of Bern, Bern, Switzerland

**Keywords:** COPD, Electrolyte disorders, Emergency, Potassium, Sodium

## Abstract

**Background:**

Electrolyte disorders are common in the emergency department. Hyponatremia is known to be associated with adverse outcome in community-acquired pneumonia (CAP) and acute exacerbation of chronic obstructive pulmonary disease (AECOPD). No studies investigating the prevalence and influence of hypernatremia or potassium disorders in patients with AECOPD exist.

**Methods:**

In this retrospective cohort analysis, the prevalence of sodium and potassium disorders was investigated in patients with AECOPD presenting to an emergency department (ED) between January 1^st^ 2017 and December 31^st^ 2018 and compared to all ED patients with electrolyte measurements and patients presenting with CAP. Exclusion criteria were age younger than 18 years, written or verbal withdrawal of consent and outpatient treatment. Additionally, the influence of dysnatremias and dyskalemias on outcome measured by ICU admission, need for mechanical ventilation, length of hospital stay, 30-day re-admission, 180-day AECOPD recurrence and in-hospital mortality and their role as predictors of disease severity measured by Pneumonia Severity Index (PSI) were investigated in patients with AECOPD.

**Results:**

Nineteen point nine hundred forty-eight ED consultations with measurements of sodium and potassium were recognized between January 1^st^ 2017 and December 31^st^ 2018 of which 102 patients had AECOPD. Of these 23% had hyponatremia, 5% hypernatremia, 16% hypokalemia and 4% hyperkalemia on admission to the ED. Hypo- and hypernatremia were significantly more common in patients with AECOPD than in the overall ED population: 23 versus 11% (*p* = 0.001) for hypo- and 5% versus 0.6% (*p* < 0.001) for hypernatremia. In the logistic regression analysis, no association between the presence of either sodium or potassium disorders and adverse outcome were found.

**Conclusion:**

Dysnatremias and dyskalemias are common in patients with AECOPD with as many as 1 in 5 having hyponatremia and/or hypokalemia. Hypo- and hypernatremia were significantly more common in AECOPD than overall. No significant association was found for dysnatremias, dyskalemias and adverse outcomes in AECOPD.

## Background

Disorders of serum sodium and potassium are common in hospitalized patients as well as in patients presenting to the emergency department (ED) [1, 2]. In terms of sodium, hypo- as well as hypernatremia are known independent predictors for mortality in critically ill patients [3, 4]. For a long time, sodium disorders were considered markers of the severity of an underlying disease, but evidence is growing that they themselves have deleterious effects on physiologic functions [5–8].

Dyskalemias are also commonly encountered in the ED as well as in hospitalized patients [1, 9–11]. It was recently stated that the risks of hypokalemia were probably underestimated and that they are comparable to or even larger than those of hyperkalemia [12]. 

Chronic obstructive pulmonary disease (COPD) is a chronic airway disease affecting approximately 5% of the population causing significant morbidity and mortality [13, 14]. During recent years, evidence grew that electrolyte disorders, especially hyponatremia might have an impact on the course and prognosis of pneumonia [15–19].

Unlike pneumonia, there is a lack of studies on the association of COPD with disorders of serum sodium and potassium, although the theoretical basis for it is broad:

Hypokalemia may be a complication of AECOPD due to decreased nutritional intake in acute illness or increased renal loss, especially in case of concomitant decompensated heart failure [12]. Additionally, it is reported, that infections to the lung, such as pneumonia, might lead to syndrome of inappropriate antidiuresis (SIAD) and thus hyponatremia [20]. Hypernatremia is always associated with hyperosmolality and thus, increased sense of thirst. It usually only develops in patients who either have a disturbed thirst sensation, no access to free water or are unable for to drink autonomously [21, 22]. Reduced general state of health in acute illness such as severe pneumonia or severe exacerbation of COPD might lead to impairment of thirst sensation for example due to fever, confusion or inflammation. Furthermore, patients might suffer from weakness or even immobility limiting their free access to water even with intact thirst sensation. Both conditions might increase the risk for hypernatremia in these patients.

Knowledge of the prevalence and relevance of dysnatremias and dyskalemias in patients with AECOPD could help to create awareness and new strategies to further influence outcome of patients with COPD in a favorable way.

In the present study, we aimed to investigate the prevalence and influence on outcome of hypo-/hypernatremia and hypo-/hyperkalemia in patients presenting to the ED with a diagnosis of AECOPD.

## Materials and methods

### Study design, aim and patient selection

In this retrospective cohort analysis, all patients admitted to the Department of Emergency Medicine of the Buergerspital Solothurn between January 1st 2017 until December 31st 2018 with measurements of serum sodium or serum potassium on admission were eligible for analysis. The Department of Emergency Medicine is a large interdisciplinary ED with approximately 35.000 annual consultations. Exclusion criteria were age younger than 18 years, written or verbal withdrawal of consent and outpatient treatment. Hyponatremia was defined as serum sodium < 135 mmol/l, hypernatremia as serum sodium > 145 mmol/l, hypokalemia as serum potassium < 3.5 mmol/l and hyperkalemia as serum potassium > 5.0 mmol/l on admission.

The aim of the study was to investigate the prevalence of dysnatremias and dyskalemias in patients with AECOPD in comparison to the overall ED population and patients with CAP. Data on electrolyte disorders in patients presenting to the ED with CAP had been investigated in the same patient collective and were recently published by our group [19]. Therefore, patients with CAP were chosen as a control group for this study on AECOPD. ICU admission, need for mechanical ventilation, length of hospital stay, 30-day re-admission, 180-day AECOPD recurrence and in-hospital mortality were assessed in patients with AECOPD in order to investigate the influence of sodium and potassium disorders on patient outcome. Finally, the role of dysnatremias and dyskalemias as predictors of disease severity was evaluated by assessing Pneumonia Severity Index (PSI) in patients with AECOPD.

### Data collection

Patients' charts were screened for a diagnosis of COPD and AECOPD was classified on basis of clinical criteria as follows: acute or subacute worsening of dyspnea (≥ 5 on a visual analogue scale that ranges from 0 to 10) sometimes but not necessarily accompanied by increased cough, sputum volume and/or sputum purulence [23]. Of all patients with AECOPD, who were hospitalized, we gathered data concerning age, sex, FEV1, GOLD stage [24], Pneumonia-Severity-Index (PSI) on admission, on-admission medication, clinical data including vital signs), serum sodium and potassium on admission, serum osmolality, urine chemistry, IMC or ICU admission, need for mechanical ventilation, length of hospital stay, 30-day re-admission, 180-day AECOPD recurrence and in-hospital mortality. Chart reviews were performed by the same persons (GL, SH, RG, SR). In order to compare prevalence rates of disorders of serum sodium and potassium in patients with AECOPD to patients with community-acquired pneumonia, data were compared to a patient collective of a previously published study [19].

### Statistical analysis

Statistical analysis was performed according to a previously published study on electrolyte disorders in patients presenting to the ED with CAP from the same database [19]. After completion of data collection, outliers (> 95% confidence interval) were reconfirmed or corrected. Data were exported to a statistical software package (SPSS for Windows, version 23; SPSS Inc; Chicago, IL) for analysis. Continuous data are presented as median and interquartile ranges or as mean and standard deviation (± SD). Distribution of continuous variables were assessed using normal plots and logarithm transformation when appropriate. Categorical data are presented as absolute counts and percent. Between-group comparisons of continuous variables were performed using one-way ANOVA and the Bonferroni test for post-hoc comparisons. Categorical variables were compared using the χ2 test or Fisher’s exact test. Logistic regression was used to explore the association of categories of serum sodium and potassium with length of stay, death, ICU referral, need for mechanical ventilation, 30-day readmission and 180-day AECOPD recurrence. In order to adjust for confounders, multivariable regression analysis was used by entering a set of predefined covariates (i.e. other present electrolyte disorders of sodium and potassium) in the model. A two-sided *p*-value < 0.05 was considered statistically significant.

### Ethical considerations

The study was approved by the local ethics committee, the Ethikkommission Nordwest- und Zentralschweiz and need for individual informed consent was waived by the committee due to the retrospective design (www.eknz.ch; project-ID: 2020–02,434). There was no patient or public involvement in the design, conduct, reporting or dissemination of this research. There was no funding for this study.

## Results

During the study period from January 1^st^ 2017 to December 31^st^ 2018, 64.713 ED consultations (2017: 31.311, 2018: 33.402) were reported. Measurements of serum sodium and potassium were available for a total of 19.948 consultations. Mean age of patients was 60 years (SD 21) and 50.5% of patients were women. 41.3% of patients were treated as outpatients. Baseline characteristics of the study population are summarized in Table 1.

**Table 1:** Baseline characteristics of the study population.

	**Non-AECOPD**	**AECOPD**	***p*-value**
**Number of patients**	19,846	102	n/a
**Age (years)**	59.62 (± 21)	73 (± 10.2)	< 0.001
**Men**	9834 (49.6%)	47 (46.1%)	0.68
**Serum Sodium (mmol/l)**	138.31 (± 3.6)	136.60 (± 6.9)	0.34
**Serum Potassium (mmol/l)**	3.93 (± 0.4)	3.99 (± 0.55)	0.8
**Serum Creatinine (umol/l)**	85.38 (± 51.26	101.57 (± 56.18)	0.04
**LOS (days)**	3.78 (± 5.0)	7.68 (± 6.54)	< 0.001
**Number of patients with traumatic injury on admission**	742 (3.7%)	0	n/a
**Death**	320 (1.6%)	12 (12%)	n/a

*AECOPD* acute exacerbation of COPD, *LOS* Length of Stay, *n/a* not applicable

### Baseline characteristics of patients with AECOPD

One hundred two patients were identified with AECOPD on admission to the ED. Mean age of patients with AECOPD was 73 years (SD 20) and 54% were women. Length of stay was 7 days (SD 5). GOLD stages were known for 71 of 102 patients (70%) and were as follows: 12 patients (17%) had COPD GOLD stage 1, 29 (41%) stage 2, 17 (24%) stage 3 and 13 (18%) stage 4. FEV 1 was available for 56 patients (55%) and median FEV1 was 52% (41 – 64). 9 patients (9%) needed ICU admission for invasive (5%) or non-invasive (4%) ventilation. 180-day AECOPD-recurrence occurred in 23 patients (23%). 12 patients (12%) died during hospitalization.

### Electrolyte disorders in patients with AECOPD

Mean serum sodium in patients with AECOPD was 137 mmol/L (SD 3.6) while mean serum potassium was 4.0 mmol/L (SD 0.5). 23 patients (23%) had hyponatremia on admission to the ED, while 5 (5%) had hypernatremia. Hypokalemia was present in 16 patients (16%) and hyperkalemia in 4 (4%). Compared to patients without AECOPD, hyponatremia was significantly more common (23 versus 11%; *p* = 0.001) and so was hypernatremia (5% versus 0.6%; *p* < 0.001) in patients presenting to the ED with AECOPD. No significant difference could be found in the prevalence of hypokalemia (16 versus 12%; *p* = 0.25) and hyperkalemia (4 versus 2%; *P* = 0.198).

### Comparison of prevalence rates of sodium and potassium disorders between patients with AECOPD and Community-acquired pneumonia

**Fig. 1:**
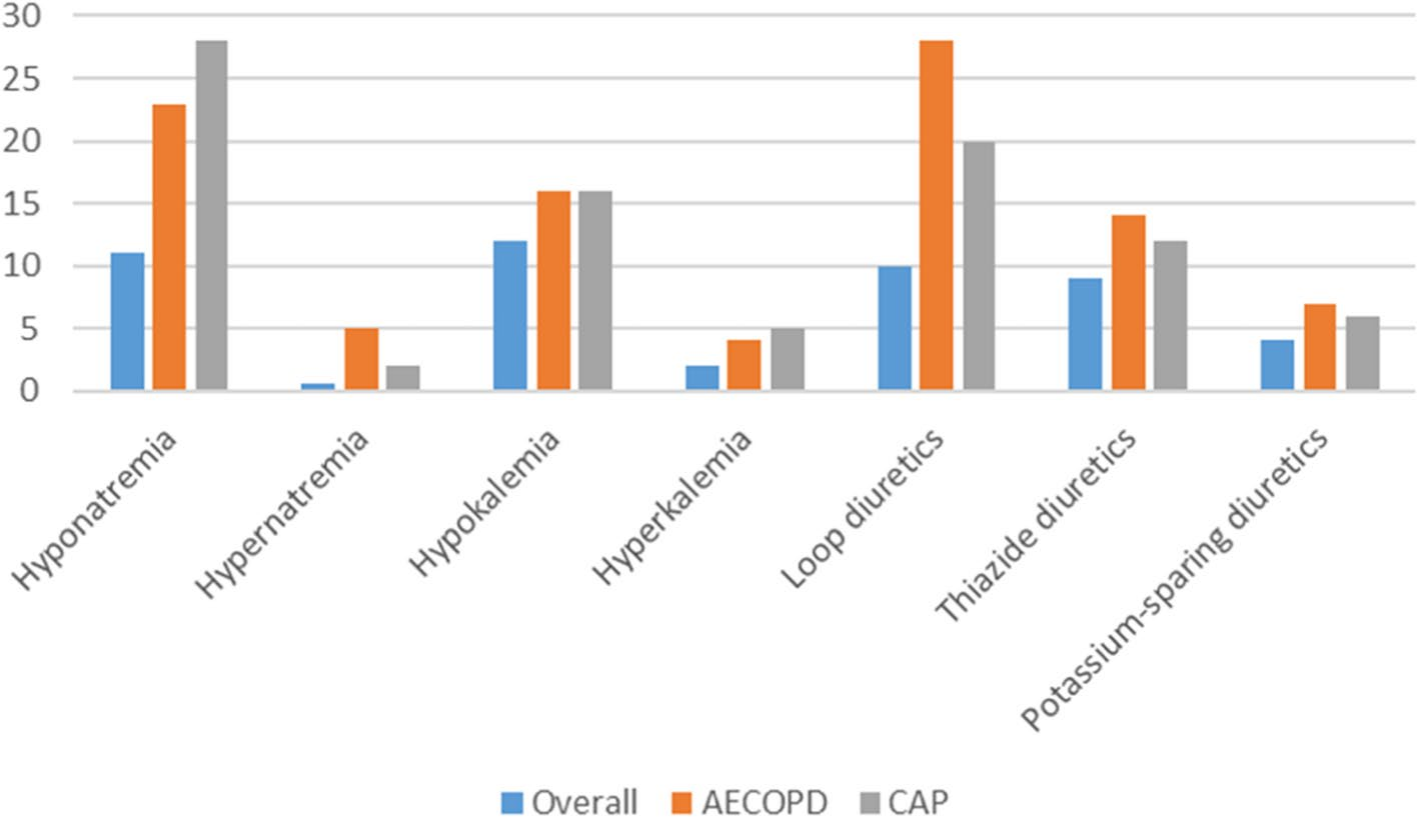
Prevalence rates for disorders of serum sodium and diuretics for patients overall, patients with acute axacerbation of COPD and Community-acquired pneumonia (CAP). Data on CAP was included from a recent publication of our study group investigating the same patient collective [19]

Prevalence rates for hyponatremia (23 versus 28%, *p* = 0.33) as well as hypernatremia (5 versus 2%, *p* = 0.09) were similar comparing patients with AECOPD to those with Community-acquired pneumonia. Also, no difference was found concerning the prevalence of hypokalemia (16 versus 16%, *p* = 0.98) as well as hyperkalemia (4 versus 5%, *p* = 0.81). Figure 1 gives the prevalence rates of electrolyte disorders as well as diuretic medication for patients overall, with AECOP as well as with community-acquired pneumonia.

### Disorders of serum sodium and influence on outcome of patients with AECOPD

In the regression analysis, neither disorders of serum sodium nor of serum potassium were associated with need for ICU treatment or invasive or non-invasive mechanical ventilation in patients with AECOPD. Additionally, dysnatremias and dyskalemias were not found to be predictive for 30-day readmission to the hospital. No association was found for sodium or potassium disorders with in-hospital mortality of patients with AECOPD. Detailed results for the regression analysis are given in Table 2.

**Table 2:** Results of the regression analysis concerning on-admission disorders of serum sodium and potassium with respect to various outcomes in patients with AECOPD

	**Hyponatremia**	**Hypernatremia**	**Hypokalemia**	**Hyperkalemia**
**ICU treatment**				
Regression coefficient	18.944	18.852	-0.336	1.273
Standard Error	17,974.834	17,974.834	1.106	1.220
*p*-value	0.999	0.999	0.761	0.297
**Invasive/Non-invasive Ventilation**				
Regression coefficient	-18.578	-18.578	-18.469	-18.469
Standard Error	8380.814	17,974.843	10,048.243	20,096.485
*p*-value	0.998	0.999	0.999	0.999
**30 day re-admission**				
Regression coefficient	0.827	0.069	0.480	-19.934
Standard Error	0.529	0.004	0.602	20,096.485
*p*-value	0.118	0.952	0.425	0.999
**In-hospital mortality**				
Regression coefficient	-0.374	0.591	-0.734	0.875
Standard Error	0.821	1.173	1.087	1.203
* p*-value	0.649	0.615	0.499	0.467

## Discussion

In the present study, we aimed to investigate the prevalence of disorders of serum sodium and potassium as well as their influence on outcome in patients with AECOPD admitted to the emergency department. Moreover, we compared the prevalence of electrolyte disorders to the overall ED population and a collective of patients admitted with CAP.

Hyponatremia as well as hypokalemia were common findings in patients with AECOPD with prevalence rates of 23 and 16%, respectively. The prevalence of hypernatremia was 5% in AECOPD, appearing intriguingly high for non-critically ill patients. Interestingly, the prevalence rates were comparable to patients with Community-acquired pneumonia, which were published previously [19].

Our finding, that hyponatremia is quite common in patients with AECOPD, stands in line with previous investigations, however, absolute numbers were relevantly higher in the present study [25, 26]. To the best of our knowledge, this was the first study investigating the prevalence rate of hypernatremia as well as dyskalemias in patients with AECOPD.

The causes for the high prevalence of hyponatremia might be explained by several reasons: On the one hand, several lung diseases are known to be strongly associated with the Syndrome of Inadequate ADH secretion (SIADH) [20]. On the other hand, patients with COPD often suffer from plenty of comorbidities requiring medications such as diuretics, which are closely associated with electrolyte disorders [2, 27]. The same hypothesis might be true for the high prevalence of hypokalemia. Only recently, hyponatremia was identified as an independent predictor of ED revisits of patients suffering from AECOPD [28].

In the present study, we could not find a statistically significant association of electrolyte disorders with adverse outcome in terms of need for ICU treatment, invasive or non-invasive ventilation, 30-day re-admission rate or in-hospital mortality in patients with AECOPD. However, the case number might potentially be low and the study underpowered to detect so.

The present study is limited by its retrospective design and hence relevant information on the cases that was not assessed on admission could not be retrieved for further investigation. Additionally, the absolute case number of AECOPD might be too low to definitely exclude an adverse impact of disorders of serum sodium and potassium on the outcome of patients. Because of the high prevalence rates found in the present study, further investigations on this important topic are wanted.

## Conclusion

In the present study, we found a high prevalence of hyponatremia as well as hypokalemia in patients with AECOPD with about 1 in 5 patients presenting with one of the electrolyte disorders. Prevalence rates of dysnatremias and dyskalemias were similar to patients with CAP. No association was found for disorders of serum sodium or potassium with adverse outcome in patients with AECOPD.

## Data Availability

The datasets generated and analyzed during the current study are not publicly available due to privacy and data safety but are available from the corresponding author on reasonable request.

## Abbreviations

AECOPD: Acute Exacerbation of COPD
CAP: Community-Acquired Pneumonia
COPD: Chronic Obstructive Pulmonary Disease
ED: Emergency Department
FEV1: Forced-Exspiratory Volume 1
ICU: Intensive Care Unit
IMC: Intermediate Care Unit
SIADH: Syndrome of Inappropriate Antidiuresis

## References

[1] Arampatzis S, Funk GC, Leichtle AB, Fiedler GM, Schwarz C, Zimmermann H, Exadaktylos AK, Lindner G (2013). Impact of diuretic therapy-associated electrolyte disorders present on admission to the emergency department: a cross-sectional analysis. BMC Med.

[2] Arampatzis S, Frauchiger B, Fiedler GM, Leichtle AB, Buhl D, Schwarz C, Funk GC, Zimmermann H, Exadaktylos AK, Lindner G (2012). Characteristics, symptoms, and outcome of severe dysnatremias present on hospital admission. Am J Med.

[3] Funk GC, Lindner G, Druml W, Metnitz B, Schwarz C, Bauer P, Metnitz PG (2010). Incidence and prognosis of dysnatremias present on ICU admission. Intensive Care Med.

[4] Lindner G, Funk GC, Schwarz C, Kneidinger N, Kaider A, Schneeweiss B, Kramer L, Druml W (2007). Hypernatremia in the critically ill is an independent risk factor for mortality. Am J Kidney Dis.

[5] Lenz K, Gössinger H, Laggner A, Druml W, Grimm G, Schneeweiss B (1986). Influence of hypernatremic-hyperosmolar state on hemodynamics of patients with normal and depressed myocardial function. Crit Care Med.

[6] Renneboog B, Musch W, Vandemergel X, Manto MU, Decaux G (2006). Mild chronic hyponatremia is associated with falls, unsteadiness, and attention deficits. Am J Med.

[7] Renneboog B, Sattar L, Decaux G (2017). Attention and postural balance are much more affected in older than in younger adults with mild or moderate chronic hyponatremia. Eur J Intern Med.

[8] Josiassen RC, Filmyer DM, Geboy AG, Martin DM, Curtis JL, Shaughnessy RA, Salzman A, Orlandi C (2012). Psychomotor deficits associated with hyponatremia: a retrospective analysis. Clin Neuropsychol.

[9] Marti G, Schwarz C, Leichtle AB, Fiedler GM, Arampatzis S, Exadaktylos AK, Lindner G (2014). Etiology and symptoms of severe hypokalemia in emergency department patients. Eur J Emerg Med.

[10] Singer AJ, Thode HC, Peacock WF (2017). A retrospective study of emergency department potassium disturbances: severity, treatment, and outcomes. Clin Exp Emerg Med.

[11] Jensen HK, Brabrand M, Vinholt PJ, Hallas J, Lassen AT (2015). Hypokalemia in acute medical patients: risk factors and prognosis. Am J Med.

[12] Clase CM, Carrero JJ, Ellison DH, Grams ME, Hemmelgarn BR, Jardine MJ, Kovesdy CP, Kline GA, Lindner G, Obrador GT, Palmer BF, Cheung M, Wheeler DC, Winkelmayer WC, Pecoits-Filho R (2020). Conference Participants: Potassium homeostasis and management of dyskalemia in kidney diseases: conclusions from a Kidney Disease: Improving Global Outcomes (KDIGO) Controversies Conference. Kidney Int.

[13] Centers for Disease Control and Prevention (CDC) (2012). Chronic obstructive pulmonary disease among adults--United States, 2011. MMWR Morb Mortal Wkly Rep.

[14] GBD 2015 Chronic Respiratory Disease Collaborators (2017). Global, regional, and national deaths, prevalence, disability-adjusted life years, and years lived with disability for chronic obstructive pulmonary disease and asthma, 1990–2015: a systematic analysis for the Global Burden of Disease Study 2015. Lancet Respir Med.

[15] Potasso L, Sailer CO, Blum CA, Cesana-Nigro N, Schuetz P, Mueller B, Christ-Crain M (2020). Mild to moderate hyponatremia at discharge is associated with increased risk of recurrence in patients with community-acquired pneumonia. Eur J Intern Med.

[16] Cuesta M, Slattery D, Goulden EL, Gupta S, Tatro E, Sherlock M, Tormey W, O'Neill S, Thompson CJ (2019). Hyponatraemia in patients with community-acquired pneumonia; prevalence and aetiology, and natural history of SIAD. Clin Endocrinol (Oxf).

[17] Müller M, Schefold JC, Guignard V, Exadaktylos AK, Pfortmueller CA (2018). Hyponatraemia is independently associated with in-hospital mortality in patients with pneumonia. Eur J Intern Med.

[18] Zilberberg MD, Exuzides A, Spalding J, Foreman A, Jones AG, Colby C, Shorr AF (2008). Hyponatremia and hospital outcomes among patients with pneumonia: a retrospective cohort study. BMC Pulm Med.

[19] Ravioli S, Gygli R, Funk GC, Exadaktylos A, Lindner G (2020). Prevalence and impact on outcome of sodium and potassium disorders in patients with community-acquired pneumonia: A retrospective analysis. Eur J Intern Med.

[20] Ellison DH, Berl T (2007). The syndrome of inappropriate antidiuresis. N Engl J Med..

[21] Lindner G, Funk GC (2013). Hypernatremia in critically ill patients. J Crit Care.

[22] Lindner G, Exadaktylos AK (2013). Disorders of serum sodium in emergency patients: salt in the soup of emergency medicine. Anaesthesist.

[23] Kim V, Aaron SD (2018). What is a COPD exacerbation? Current definitions, pitfalls, challenges and opportunities for improvement. Eur Respir J.

[24] Global Initiative for Chronic Obstructive Lung Disease: Pocket Guide to COPD Diagnosis, Management and Prevention: https://goldcopd.org/wp-content/uploads/2018/02/WMS-GOLD-2018-Feb-Final-to-print-v2.pdf.

[25] Chalela R, González-García JG, Chillarón JJ, Valera-Hernández L, Montoya-Rangel C, Badenes D, Mojal S, Gea J (2016). Impact of hyponatremia on mortality and morbidity in patients with COPD exacerbations. Respir Med.

[26] García-Sanz MT, Martínez-Gestoso S, Calvo-Álvarez U, Doval-Oubiña L, Camba-Matos S, Rábade-Castedo C, Rodríguez-García C, González-Barcala FJ (2020). Impact of Hyponatremia on COPD Exacerbation Prognosis. J Clin Med.

[27] Cavaillès A, Brinchault-Rabin G, Dixmier A, Goupil F, Gut-Gobert C, Marchand-Adam S, Meurice JC, Morel H, Person-Tacnet C, Leroyer C, Diot P (2013). Comorbidities of COPD. Eur Respir Rev.

[28] Tokgöz Akyıl F, Tural Önür S, Abalı H, Sökücü S, Özdemir C, Boyracı N, Kocaoğlu A, Altın S (2021). Hyponatremia is an indeopendent predictor of emergency department revisits in acute exacerbation of COPD. Clin Respir J.

